# Features of Hydrogen Reduction of Fe(CN)_6_^3−^ Ions in Aqueous Solutions: Effect of Hydrogen Dissolved in Palladium Nanoparticles

**DOI:** 10.3390/nano11102587

**Published:** 2021-09-30

**Authors:** Roman Solovov, Boris Ershov

**Affiliations:** Frumkin Institute of Physical Chemistry and Electrochemistry, Russian Academy of Science, Leninsky pr. 31-4, 119071 Moscow, Russia; ershov@ipc.rssi.ru

**Keywords:** palladium, nanoparticles, hydrogen, reduction, catalytic reaction, presaturation

## Abstract

Preliminary saturation of 2.6 nm palladium nanoparticles with hydrogen accelerates the reduction of *Fe(CN)*_6_^3−^ ions in aqueous solution three to four-fold. An analytical equation was derived describing the hydrogen saturation of palladium nanoparticles and the dependence of their catalytic activity on the hydrogen content in the metal. The specific rate constants of reduction do not depend on the content of palladium nanoparticles in the solution. A change in the temperature and pH or stirring of the solution do not affect the rate of catalytic reaction. Approaches to optimization of palladium-catalyzed reactions involving hydrogen are substantiated.

## 1. Introduction

Nanomaterials have found wide use in various branches of industry, technology, medicine, and agriculture. Much attention is paid to the production and investigation of nanomaterials based on metals, especially gold or silver. However, properties of other transition metals have also attracted ever increasing attention, and the fabrication of metals in highly dispersed states is a key challenge in the synthesis and manufacture of efficient and selective catalysts [1,2]. The increase in the surface area to volume ratio with decreasing nanoparticle size markedly enhances catalytic activity [3,4,5,6,7,8].

Aqueous solutions of noble metal nanoparticles are interesting objects for the investigation of catalyst properties. The applicability of nanoparticles of a specified size and shape and the use of highly sensitive spectrophotometric method to monitor the reaction, the state of metal particles, and reactant concentrations makes this method convenient and useful for studying the mechanism of catalytic processes. The available experience, which mainly refers to gold, confirms this point of view. In particular, the reaction between ferricyanide and thiosulfate ions was found to be strongly catalyzed by citrate-stabilized gold sols [9,10,11]. A considerable catalytic effect of spherical gold nanoparticles on the rate of reduction of hexacyanoferrate(III) by sodium borohydride in aqueous solution was reported [12]. It was shown [13] that *Au*(III) ions can be reduced by hydrogen on gold nanoelectrodes. The electron transfer reaction between hexacyanoferrate(III) ions and thiosulfate ions is known to be catalyzed by platinum nanoparticles [14,15].

Since palladium is one of the most efficient metals for catalysis, development of synthesis of *Pd* NPs of a desired size and studies of their properties form an important and promising research area [16,17,18]. The synthesis is often carried out in nonaqueous media using micellar solutions containing numerous side components and reaction products, which markedly affect the catalytic and physicochemical properties of nanoparticles [19,20,21,22]. Therefore, the search for new approaches to «chemically pure» synthesis of metal (in particular, palladium) nanoparticles as hydrosols is an important task. There are quite a few publications devoted to the dissolution and state of hydrogen in palladium metal and changes in its physical characteristics [23,24]. Hydrogen dissolved in the metal has virtually no effect on the physicochemical and catalytic properties of *Pd* NPs. Therefore, the study of the mechanisms and key regularities of catalytic reactions involving *Pd* NPs and molecular hydrogen is of prime importance. It was shown that saturation of colloidal solutions of palladium with hydrogen induces transformation of the absorption band with a maximum at 220 nm corresponding to nano-sized metal particles into a new, wider band with a maximum at 265 nm [25,26]. When hydrogen is pumped off and the solution is kept under a vacuum, the initial absorption band is restored, which attests to reversibility of the sorption-desorption process. The differences in the UV/Vis spectra definitely indicate a difference between the electronic states of these nano-sized palladium particles. Indeed, it was found that *Pd* NPs of 5.3 nm size catalyze the reduction of *Fe(CN)*_6_^3−^ ions with hydrogen in an aqueous solution. The reaction rate considerably (approximately 5.5-fold) increases if the nanoparticles are pre-saturated with hydrogen [26]. This opens up new opportunities for targeted increase in the catalytic activity and selectivity of palladium in hydrogen-involving reactions.

The aim of this work is to conduct a systematic study of the effect of dissolved hydrogen in *Pd* NPs on the kinetics and mechanism of their catalytic action towards reduction of *Fe(CN)*_6_^3−^ ions with hydrogen in an aqueous solution and to elucidate the role of the concentration of reactants and medium factors (pH, temperature, etc.) in the reaction. It is also interesting to compare the catalytic effects of palladium and other metals, which, unlike palladium, do not show the ability to accumulate [9,10,11,12,13,14,15]. The results are meant for substantiation of new approaches for optimization of catalytic reactions involving palladium.

## 2. Experimental

*Pd* NPs were obtained by photochemical reduction in water in the presence of sodium polyacrylate as a stabilizing agent [27,28].

A solution containing *[Pd(NH*_3_*)*_4_*]Cl*_2_ (Aldrich, St. Louis, MO, USA) (0.20 mM) and sodium polyacrylate (PANa, average molecular mass of 2100 Da, Fluka) (monomer unit concentration in the solution of 10.0 mM) was deaerated using an NVR-4.5D sliding vane rotary vacuum pump (Vakma) equipped with a liquid nitrogen trap at residual pressure P_res_ = 2.0 Pa. Then, the solution was placed into a quartz cell and irradiated by UV light of a low-pressure xenon flash lamp at a total flow intensity of I_UV_ = 6.0 × 10^20^ quant/s = 1.0 mE/s. As a result, the *Pd*(II) ions were completely reduced. All solutions were prepared in deionized triply distilled water (specific resistance of 17.0 Ω·m).

The catalytic reduction of *Fe(CN)*_6_^3−^ ions in aqueous solutions in the presence of *Pd* NPs was carried out by hydrogen saturation of a deaerated solution containing *K*_3_*Fe(CN)*_6_. The standard pH of the solutions was 7.3. It is known the stabilizing agent affects catalytic activity of metal nanoparticles. The activity decreases with an increase of its concentration [29,30].

The study of this effect was not the aim of this work, and therefore the concentration of water-soluble stabilizer (sodium polyacrylate) in the experiments was constant (1.00 mM).

The catalytic activity of palladium towards *Fe(CN)*_6_^3−^ reduction with hydrogen was measured in two regimes, namely with and without preliminary hydrogen saturation. In the latter case (without saturation), a solution containing *K*_3_*Fe(CN)*_6_ and *Pd* NPs was prepared. The solution was deaerated, then hydrogen was added and the *Fe(CN)*_6_^3−^ reduction kinetics was measured. In the case of pre-saturation with hydrogen, a palladium hydrosol was kept in a hydrogen atmosphere for several hours (the standard saturation time was 24 h). This resulted in *Pd* NPs being saturated with hydrogen. Different degrees of palladium «hydrogenation» were attained by conducting saturation at different hydrogen pressures in the solution.

The solution acidity was varied by adding required amounts of HClO_4_ or NaOH until the specified pH was attained. The temperature was varied in the range from 10.0 to 25.0 °C.

The kinetics of *Fe(CN)*_6_^3−^ reduction with hydrogen was measured by monitoring disappearance of the absorption band at 420 nm (*ε*_420_(*Fe(CN)*_6_^3−^) = 1.1 × 10^4^ M^−1^∙cm^−1^). The measurements were carried out on a Cary 100 Scan UV-Visible Spectrophotometer equipped with a thermostated cell holder. Cooling or heating of the cell was accomplished by built-in Peltier elements, which allow variation of the temperature of the optical cell containing the solution. The optical spectra were measured at 20.0 °C (except for experiments on the effect of temperature on the reaction rate). The kinetic curves were fitted using the Origin 2018 program. This program allows to fit the experimental curve using the equation y = A × exp(−k × t), (A and k – constants). All kinetic experiments were repeated at least three times. They were reproduced with an error of ±10%.

The nanoparticle size and shape after formation of the colloidal solutions was examined using a JEM-2100 JEOL transmission electron microscope with an accelerating voltage of 200 keV. For this purpose, a drop of the colloidal solution was applied on a copper grid (100 mesh) precoated by formvar and carbon and then excess liquid was removed by filter paper and the liquid was allowed tog completely dry.

The size of formed micelles and the ζ-potential were determined on a Delsa Nano C light-scattering instrument (Beckman Coulter, Inc., Brea, CA, USA) at the wavelength of scattered laser radiation *λ*_0_ = 658 nm. The calculations were carried out using the Delsa Nano Software program package. This method determines the average particle size from the particle size distribution derived from the time correlation functions of scattered light intensity. The same principle was utilized to measure the electrophoretic mobility of the particles, which was then converted to ζ-potential using the Smoluchowski theory and corrections for different widths of the electrical double layer.

## 3. Results and Discussion

**Figure 1:**
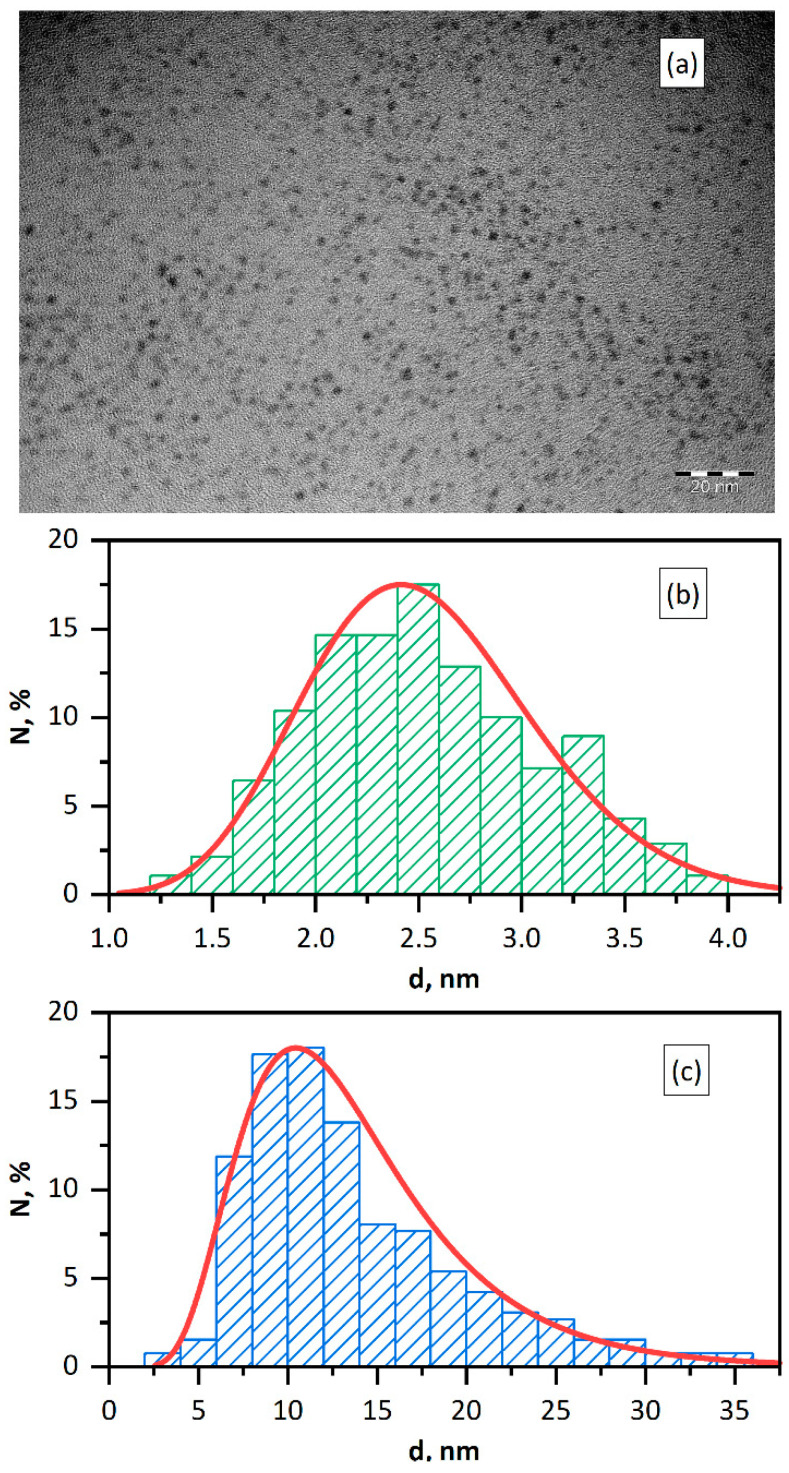
TEM image of *Pd* NPs (**a**). Size distribution of *Pd* NPs according to TEM (**b**) and DLS (**c**) data. Solution: [*Pd*^0^] = 0.20 mM, [PANa] = 10.0 mM.

The investigation results showed that the procedure of photochemical synthesis used gives reproducible and stable hydrosols of *Pd* NPs of (2.5 ± 0.6) nm size, according to transmission electron microscopy (TEM) data (Figure 1a,b). According to dynamic light scattering (DLS) data, the micelle hydrodynamic diameter is (12.4 ± 6.2) nm and zeta-potential is ζ = −(58.0 ± 4.0) mV (Figure 1c). The hydrodynamic sizes of nanoparticles are markedly larger that the sizes obtained by electron microscopy. It is caused by the fact that TEM gives the size of the metal core, while DLS measures the particle size including the stabilizing shell.

### 3.1. Kinetics and Mechanism of the Catalytic Reduction of Fe(CN)_6_^3−^ Ions with Hydrogen

The difference between the standard redox potentials of the *Fe(CN)*_6_^3^*^−^/Fe(CN)*_6_^4−^ (+360 mV) and 2*H^+^/H*_2_ (0 mV) pairs indicates the possibility of spontaneous reduction of *Fe(CN)*_6_^3−^ ions with hydrogen in aqueous solution:

$$H_{2}+2{\left[\mathrm{Fe}{\left(\mathrm{CN}\right)}_{6}\right]}^{3-}\overset{\;{\mathrm{Pd}}^{0}\;}{\to }2{\left[\mathrm{Fe}{\left(\mathrm{CN}\right)}_{6}\right]}^{4-}{+2H}^{+}\;$$

**Figure 2:**
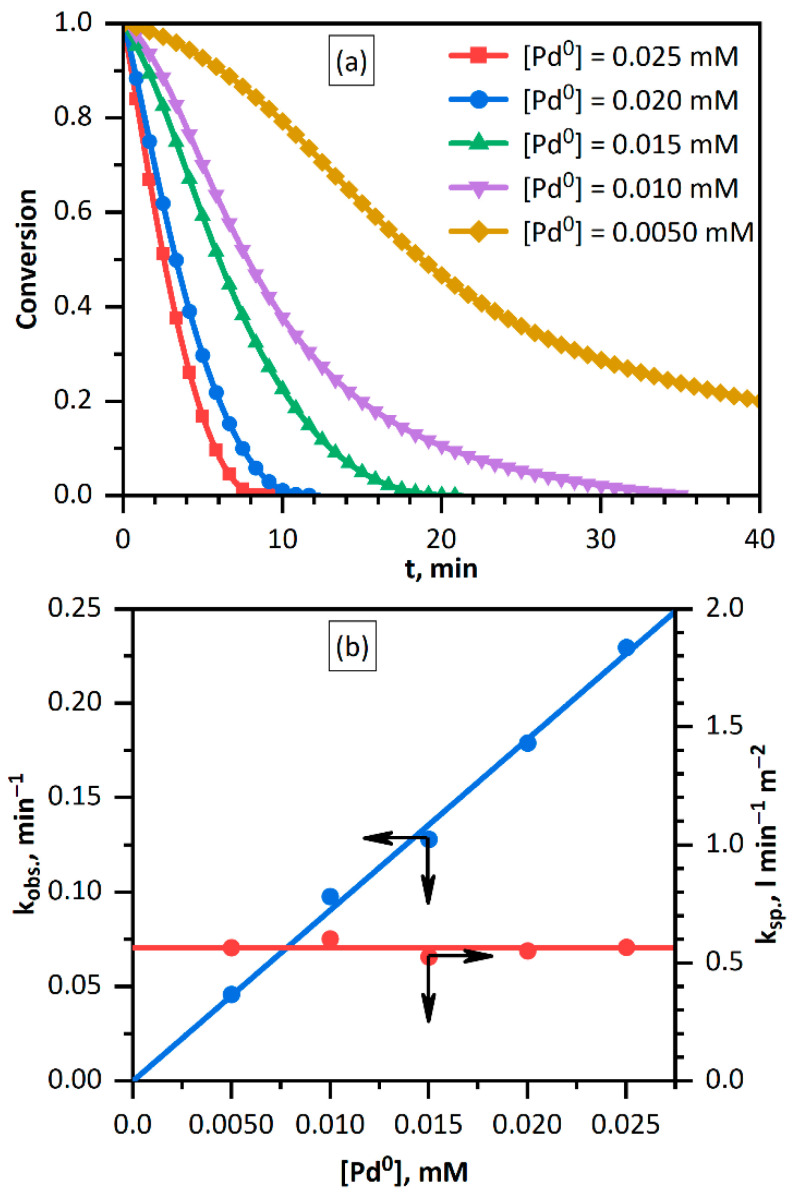
(**a**) Kinetic curves for the reduction of *Fe(CN)*_6_^3−^ ions with hydrogen in the presence of various amounts of palladium. (**b**) Observed and specific rate constants of the reduction of *Fe(CN)*_6_^3−^ ions with hydrogen vs. palladium concentration. Solution: [*Fe(CN)*_6_^3−^] = 1.00 mM, [H_2_] = 0.80 mM (p(H_2_) = 1.025 atm).

However, it was shown that the reaction proceeds very slowly (about 24 h). The introduction of *Pd* NPs sharply accelerates the reaction, and the higher the palladium concentration, the more pronounced the acceleration (Figure 2a). The decrease in the *Fe(CN)*_6_^3−^ concentration detected by the decrease in the absorbance at 420 nm follows an exponential time dependence, which attests to the first order of reaction with respect to this reagent. The initial part of the curve starts after an induction period. The observed rate constants were estimated on the initial part of the kinetic curve and exponential law is valid here. In the case of an induction period exponential fitting was made on the section of the curve after the induction period.

The rate constants of the reaction were found to follow a linear dependence on the concentration of *Pd* NPs (Figure 2b). This is typical of a heterogeneous catalytic reaction and is a consequence of linear dependence of the rate on the catalyst surface area. The specific rate constant, i.e., the constant per unit total surface area of all nanoparticles *S_tot_* was calculated in the following way. It was assumed that palladium particles have identical size with the radius *r_Pd_* and, correspondingly, each consists of *n_Pd_* palladium atoms. The number of palladium particles *N_Pd_* in the solution with the concentration [*Pd*^0^] of the palladium atoms (in mM) is determined by the formula

$$N_{\mathrm{Pd}}=\left[{\mathrm{Pd}}^{0}\right]\cdot \frac{N_{A}}{n_{\mathrm{Pd}}}\;$$

where *N_A_* is the Avogadro number. Then the specific rate constant can be calculated taking into account that

$$S_{\mathrm{tot}.}=4\cdot \pi \cdot r_{\mathrm{Pd}}^{2}\cdot N_{\mathrm{Pd}}=4\cdot \pi \cdot r_{\mathrm{Pd}}^{2}\cdot \left[{\mathrm{Pd}}^{0}\right]\cdot \frac{N_{A}}{n_{\mathrm{Pd}}}\;$$

using the equation

$$k_{\mathrm{sp}.}=\frac{k_{\mathrm{obs}.}}{S_{\mathrm{tot}.}}=\frac{k_{\mathrm{obs}.i}}{4\cdot \pi \cdot r_{\mathrm{Pd}}^{2}\cdot \left[{\mathrm{Pd}}^{0}\right]\cdot \frac{N_{A}}{n_{\mathrm{Pd}}}}\;$$

It can be seen that all parameters appearing in the equation are constants, except for *k_obs._* and [*Pd*^0^], and the *k_obs._*/[*Pd*^0^] ratio determines the relative specific rate constant *k_sp._* for the reduction of *Fe(CN)*_6_^3−^ ions with hydrogen in aqueous solution catalyzed by palladium particles. This consideration does not in any way take into account the real size distribution of nanoparticles in solution. Therefore, it is only a theoretical approach to explaining the obtained dependences.

Figure 2b shows the calculated specific rate constants for each palladium concentration. No concentration dependence is present, which confirms the adequacy of the approach used to describe the catalytic nature of palladium in the reduction of *Fe(CN)*_6_^3−^ ions with hydrogen in aqueous solution.

In a classical heterogeneous catalytic process, several mass transfer-related steps are distinguished. This is the external diffusion, that is, diffusion of reactants towards the particle surface, and the internal diffusion, that is, diffusion from the particle surface into the particle interior. In order to find out whether the catalytic process occurs in the external diffusion region, we carried out reduction catalyzed by *Pd* NPs at different solution acidities.

The dissociative adsorption and ionization of hydrogen on the nanoparticle surface gives rise to H^+^ cations. According to Fick’s law, their desorption and diffusion away from the surface are proportional to the concentration gradient of these cations in the direction «nanoparticle surface → solution bulk». The increase in the H^+^ concentration in the solution bulk decreases this gradient and retards the withdrawal of the H^+^ cations away from the surface and, hence, retards the desorption from the surface. This, in turn, slows down the hydrogen discharge-ionization on the palladium nanoparticle surface. Thus, the external diffusion of the reaction products away from the surface is the rate-limiting step. As expected, upon alkalization, the opposite trend is observed; the increase in the H^+^ concentration in the solution would finally accelerate the whole process.

**Figure 3:**
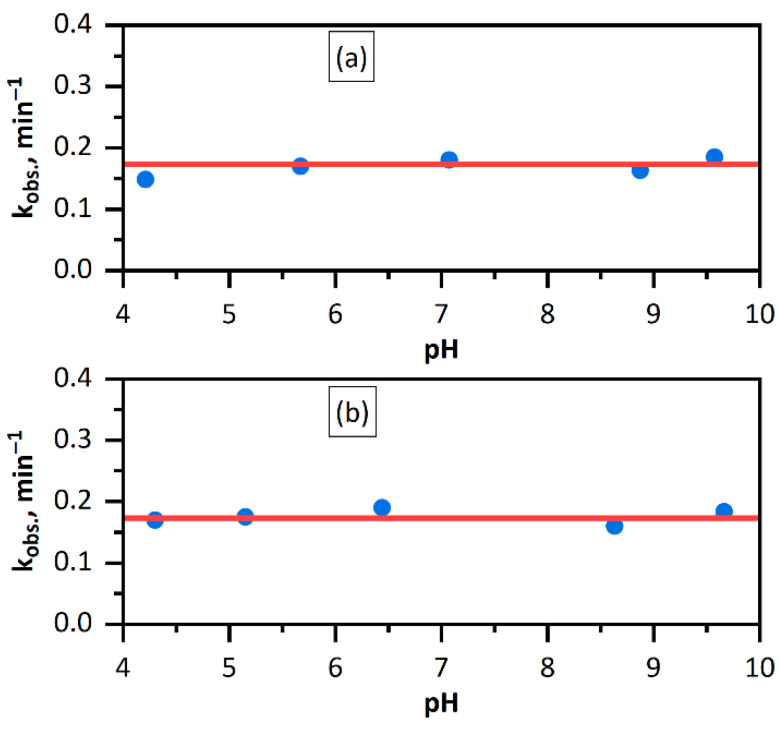
Observed rate constants of the reduction of *Fe(CN)*_6_^3−^ ions with hydrogen vs. pH of the solution in non-stirring (**a**) and stirring (**b**) regimes. Solution: [*Fe(CN)*_6_^3−^] = 1.00 mM, [*Pd*^0^] = 0.020 mM, [H_2_] = 0.80 mM (p(H_2_) = 1.025 atm).

The effect of the solution acidity was investigated by addition of *HClO*_4_ (down to pH = 4.0) or *NaOH* (up to pH = 10.0) to the initial solution. As can be seen in Figure 3, the rate of *Fe(CN)*_6_^3−^ reduction with hydrogen does not depend on the pH. Thus, it can be concluded that neither desorption of protons from the palladium nanoparticle surface nor the proton diffusion from the surface into the solution interior is not the rate-limiting step. Being a classical hydrogen microelectrode, the palladium nanoparticle does not have a clear-cut pH function.

At high nanoparticle surface coverages by polyelectrolyte and/or reaction products, a change in the concentration gradient of some ions does not significantly affect the process rate. Therefore, pH dependence was studied in two regimes, one with and the other without stirring of the solution. It was found that the onset of stirring switches on a rapid turbulent flow. Coinciding rate constants of the reduction of *Fe(CN)*_6_^3−^ ions with hydrogen with and without stirring (see Figure 3) indicate that the reaction is not rate-limited by any diffusion processes. In a study of the kinetics of *AuCl*_4_*^−^* reduction with hydrogen catalyzed by gold nanoparticles, the reaction rate was found to be dependent on the solution acidity; in other words, a gold microelectrode has a clear-cut pH-function. The data presented here (Figure 3) attest to the absence of this pH-function in palladium microelectrodes (*Pd* NPs). It is necessary to note that a change pH in the same range affects markedly on the catalytic properties of gold nanoparticles in reduction of Au^3+^ ions by hydrogen [13]. Consequently, the mechanism of hydrogen discharge and electron transfer in redox reactions differs significantly for the nanoparticles of these metals in water.

### 3.2. Effect of the Amount of Hydrogen Dissolved in Pd NPs

Particular attention was devoted to the effect of the amount of hydrogen dissolved in *Pd* NPs and hydrogen concentration in the solution on the reduction rate. The process kinetics was studied in two regimes. In the first one, the solution containing *Fe(CN)*_6_^3−^ ions and *Pd* NPs was saturated with hydrogen and the kinetic measurements were immediately carried out. In the second regime, the palladium hydrosol was kept in a hydrogen medium for five days before the reaction and then mixed with other components and the kinetics was measured. During the five days, with stirring under a moderate hydrogen excess pressure, the palladium hydrosol was saturated with hydrogen and hydrogen was dissolved in metal nanoparticles (this will be designated as «hydrogenated» palladium). The considerable difference between the UV/Vis spectra of the catalysts obtained by these procedures points to different electronic states of these forms of nano-sized palladium. Thus, in the reactions conducted in the two regimes (with and without saturation), two different materials served as the catalyst: *Pd* NPs and hydrogenated *Pd* NPs.

A minor change in the zeta-potential of *Pd* NPs after hydrogen saturation with respect to the initial value (before hydrogenation, ζ = −(58.0 ± 4.0) mV; after hydrogenation, ζ = −(47.3 ± 9.0) mV) attests to a change in the state of metal surface. However, relatively high absolute value (more than 40 mV) is indicative of high aggregative and sedimentation stability of the hydrosol. According to transmission electron microscopy, the palladium nanoparticle size and size distribution remain the same after hydrogenation. This is also confirmed by dynamic light scattering.

**Figure 4:**
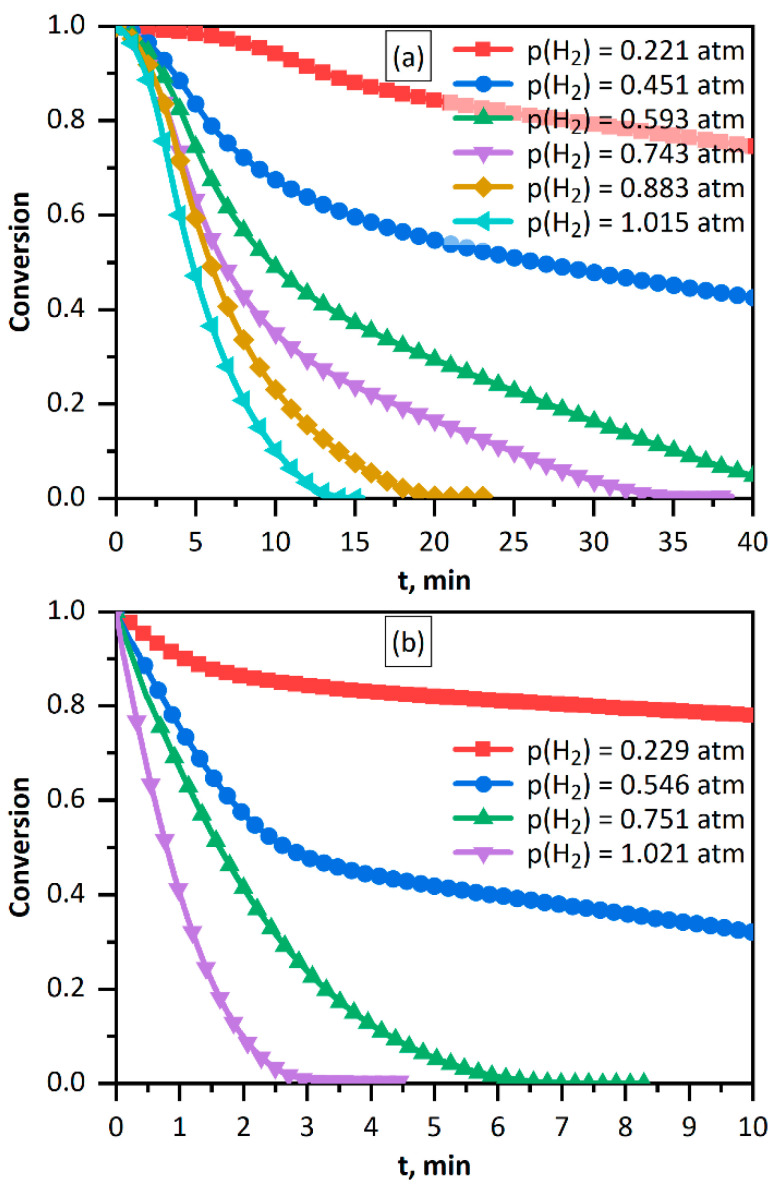
Kinetic curves for *Fe(CN)*_6_^3−^ reduction with hydrogen. Hydrogen pressure above the solutions given in legend. Without (**a**) and with (**b**) preliminary saturation of palladium hydrosol with hydrogen. Solutions: [*Fe(CN)*_6_^3−^] = 1.00 mM, [*Pd*^0^] = 0.020 mM.

Figure 4 shows the kinetic curves for *Fe(CN)*_6_^3−^ reduction with hydrogen depending on the hydrogen pressure above the solution. First of all, attention is attracted by the fact that the reduction rate is markedly higher on the «hydrogenated» palladium than on «non-hydrogenated» palladium. For equal catalyst and reactant concentrations, the rates differ approximately 3–4-fold. Hydrogen saturation of palladium and hydrogen removal from palladium upon solution evacuation are fairly slow and last for tens of minutes [25,26,31]. This means that the *Fe(CN)*_6_^3−^ reduction with hydrogen conducted within a few to ten minutes involves *Pd* NPs in the initial chemical form (either «hydrogenated» or not). It is noteworthy that the concentration of hydrogen stored in «hydrogenated» palladium is too low to affect the reduction. When the *Pd*^0^ concentration was 0.020 mM and the *Pd*^0^*/H*_2_ ratio was approximately 0.35 to 0.65, the dissolved *H*_2_ content was only about 0.010 M. This is incommensurably low with respect to the electron equivalent needed for the reduction of 1.0 mM *Fe(CN)*_6_^3−^.

The curves for the case of the non-hydrogenated palladium clearly show an induction period the duration of which increases with decreasing hydrogen content. However, no induction period is present in the curves recorded with hydrogenated palladium. This finding suggests the presence of an equilibrium for non-hydrogenated palladium, which is established during the induction period. This can probably be attributed to the dissociative adsorption, which takes place on nanoparticle surface.

Although the dissociative adsorption is considered to have zero activation energy, when the hydrogen contact with the metal surface is hampered, excess energy is required to overcome some energy barrier. In the palladium hydrosol used in this work, the contact can be hampered because of nanoparticle surface coating by the stabilizing polyelectrolyte and/or the presence of electrical double layer. Furthermore, the presence of multicharged *K*_3_*Fe(CN)*_6_ electrolyte may cause a considerable rearrangement of the electrical double layer and charge reversal of the ion-stabilized micelles in solution. These factors complicate the processes that occur on the particle surface and complicate the contact with the particle.

The assumption of the dissociative adsorption is supported by disappearance of the induction period on going to experiments involving hydrogenated palladium (Figure 4). In this case, palladium does contain hydrogen and equilibrium has already established. The appearance of the induction period in the kinetic curves in the presence of «non-hydrogenated» palladium apparently confirms the fact that dissociative adsorption and associative desorption are equilibrium processes with slowly established equilibrium.

**Figure 5:**
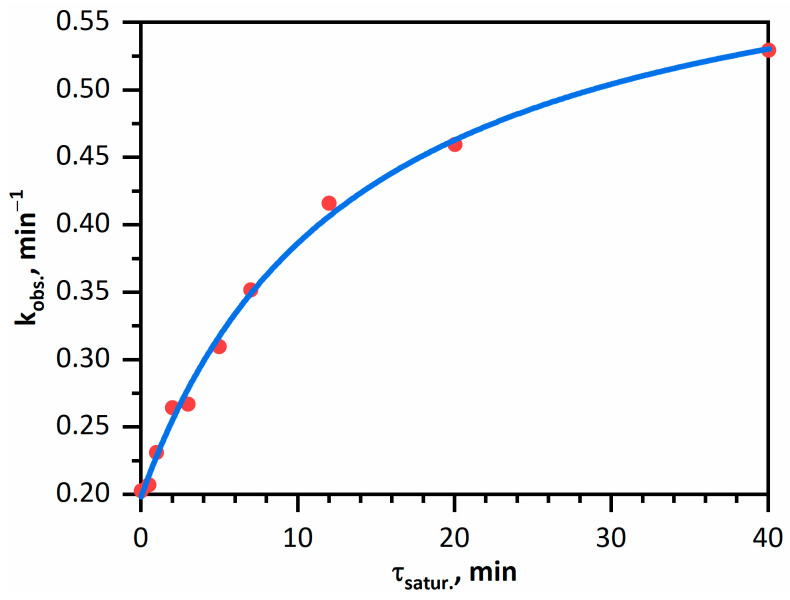
Observed relative rate constant of *Fe(CN)*_6_^3−^ reduction with hydrogen vs. time of palladium saturation with hydrogen. The dots correspond to the experiment and the curve shows calculations. Solution: [*Fe(CN)*_6_^3−^] = 1.00 mM, [*Pd*^0^] = 0.020 mM, [H_2_] = 0.80 mM (p(H_2_) = 1.025 atm).

The substantially slower removal of hydrogen from «hydrogenated» palladium even with evacuation of the solution (tens of minutes) compared with the rate of *Fe(CN)*_6_^3−^ reduction with hydrogen (minutes) can be used to investigate the effect of the degree of palladium saturation with hydrogen on its catalytic activity. The effect was evaluated by the method of relative rate constants. The method consisted in measurement of the observed reaction rate constants in the presence of *Pd* NPs with variable degree of hydrogen saturation, with vigorous stirring for various periods of time. Figure 5 shows the dependence of the rate constant of *Fe(CN)*_6_^3−^ reduction with hydrogen catalyzed by palladium samples with different degrees of «hydrogenation».

The saturation of palladium with hydrogen and the subsequent hydrogen discharge on palladium can be represented as follows:

$$H_{2\;\mathrm{ads}.}{\;+\;2\mathrm{Pd}}^{0}\overset{\;K\;}{↔}{2\mathrm{Pd}}^{0}-H\overset{\;\;k^{\prime }\;\;}{\to }{2\mathrm{Pd}}^{0}{\;+\;2H}^{+}{+\;2e}^{-}\;H_{2\;\mathrm{soln}}{\;+\;2\mathrm{Pd}}^{0}\overset{\;k\prime \prime \;}{\to }{2\mathrm{Pd}}^{0}{\;+\;2H}^{+}{+\;2e}^{-}$$

where *K* is the equilibrium constant of dissociative adsorption; *k′* is the rate constant of *Fe(CN)*_6_^3−^ reduction by hydrogen from palladium hydride; *k″* is the rate constant of *Fe(CN)*_6_^3−^ reduction by hydrogen from solution on nanoparticles surface. Considering the experimentally established dependences, it can be stated that the electron transfer to *Fe(CN)*_6_^3−^ is fast. Then the observed rate constant of *Fe(CN)*_6_^3−^ reduction can be written as follows:

$$k_{\mathrm{obs}.}=k^{\prime }+k^{\prime \prime }=\alpha \cdot \theta_{H}+k^{\prime \prime }\;$$

where *k_obs._* is the observed rate constant of *Fe(CN)*_6_^3−^ reduction, *α* is an arbitrary proportionality factor, and *θ_H_* is the coverage of the nanoparticle surface with hydrogen atoms (degree of dissociative adsorption), which can be expressed as

$$\theta_{H}=\frac{K\cdot \left[H_{2\;\mathrm{ads}.}\right]}{1+K\cdot \left[H_{2\;\mathrm{ads}.}\right]}\;$$

The concentration of the adsorbed hydrogen on the surface of the [*H*_2 *ads.*_] species is proportional to the time of hydrosol saturation with hydrogen (*τ_satur._*). Then the expressions for the observed rate constant *k_obs_* and the degree of dissociative adsorption *θ_H_* can be converted to

$$k_{\mathrm{obs}.}=\alpha \cdot \theta_{H}+k^{\prime \prime }=\alpha \cdot \frac{K\cdot \left[H_{2\;\mathrm{ads}.}\right]}{1+K\cdot \left[H_{2\;\mathrm{ads}.}\right]}+k^{\prime \prime }=\alpha \cdot \frac{{K\cdot \beta \cdot \tau }_{\mathrm{satur}.}}{1+K\cdot \beta \cdot \tau_{\mathrm{satur}.}}+k\prime \prime \;$$

where *β* is the proportionality factor.

The obtained experimental dependence is well described by this equation with the following parameters: *α* = 0.45, *K × β* = 0.0012. It is noteworthy that in essence, this expression follows from the Langmuir equation for monomolecular adsorption. The adsorption is complicated by its polymolecular nature, nonequivalence of the adsorption sites (the nanoparticle has an anisotropic surface), and diffusion of hydrogen from the surface inside the metal cores.

The effect of hydrogen concentration on the *Fe(CN)*_6_^3−^ reduction rate was studied by changing the hydrogen pressure above the solution with allowance for the Henry’s law. According to the Sieverts’ law, upon gas dissolution in the metal, the concentration of atomic hydrogen formed in the reaction is described by the equation

$$K_{S}=\frac{{\left[H\right]}_{\mathrm{Pd}}^{2}}{p\left(H_{2}\right)}=\frac{{\left[H\right]}_{\mathrm{Pd}}^{2}}{K_{H}\cdot {\left[H_{2}\right]}_{\mathrm{soln}}}\;$$

where *K*_S_ is the equilibrium constant in the Sieverts law, *[H]_Pd_* is the concentration of hydrogen atoms in palladium, *[H*_2_*]_soln_* is the concentration of molecular hydrogen in aqueous solution, and *K_H_* is the Henry constant. Hence, the concentration of hydrogen atoms in the metal can be expressed in the following way:

$${\left[H\right]}_{\mathrm{Pd}}={\left(K_{S}{\cdot K}_{H}\cdot {\left[H_{2}\right]}_{\mathrm{soln}}\right)}^{½}\;$$

**Figure 6:**
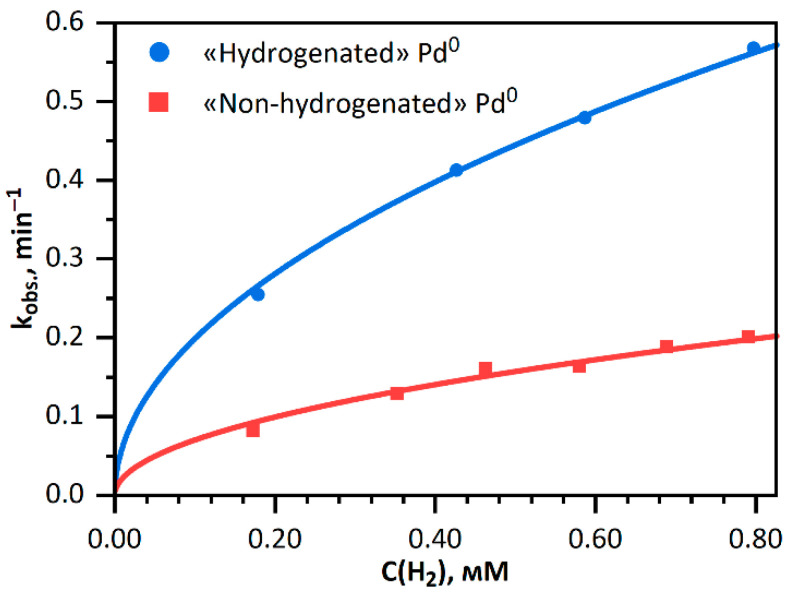
Observed rate constant of *Fe(CN)*_6_^3−^ reduction with hydrogen in the presence of «hydrogenated» palladium «non-hydrogenated» palladium vs. hydrogen concentration in the solution. The dots correspond to the experiment and the curve shows calculations. Solution: [*Fe(CN)*_6_^3−^] = 1.00 mM; [*Pd*^0^] = 0.020 mM.

It can be seen from Figure 6 that the rate constant of the *Fe(CN)*_6_^3−^ reduction with hydrogen in the presence of hydrogenated palladium increases with increasing concentration of hydrogen in solution. The pattern of dependence is well described by an equation with ½ power of the hydrogen concentration in solution:

$$k_{\mathrm{obs}.}\;\sim \;\gamma \cdot {\left({\left[H_{2}\right]}_{\mathrm{soln}}\right)}^{½}\;$$

where *γ* is the proportionality factor.

Since the parameters contained in the proportionality factor *γ* are unknown, the calculation of the absolute numerical values of the equilibrium constants in the Sieverts’ equation is difficult. However, comparison of the proportionality factors *γ* for hydrogenated and non-hydrogenated palladium reveals an eight-fold difference of the equilibrium constants:

$${\left(\frac{\gamma_{\;«\mathrm{hydr}.»}}{\gamma_{\;«\mathrm{non}-\mathrm{hydr}.»}}\right)}^{2}\;=\;{\left(\frac{{\left(K_{S\;«\mathrm{hydr}.»}{\cdot K}_{H}\right)}^{½}}{{\left(K_{S\;«\mathrm{non}-\mathrm{hydr}.»}{\cdot K}_{H}\right)}^{½}}\right)}^{2}\;=\;\frac{K_{S\;«\mathrm{hydr}.»}}{K_{S\;«\mathrm{non}-\mathrm{hydr}.»}}\;=\;8.0\;$$

The equilibrium constant of dissociative adsorption described by the Sieverts’ law proves the possibility of this process and indicates the degree to which it occurs. The juxtaposition of these rate constants points to fundamental differences between the two materials (i.e., hydrogenated and non-hydrogenated palladium).

**Figure 7:**
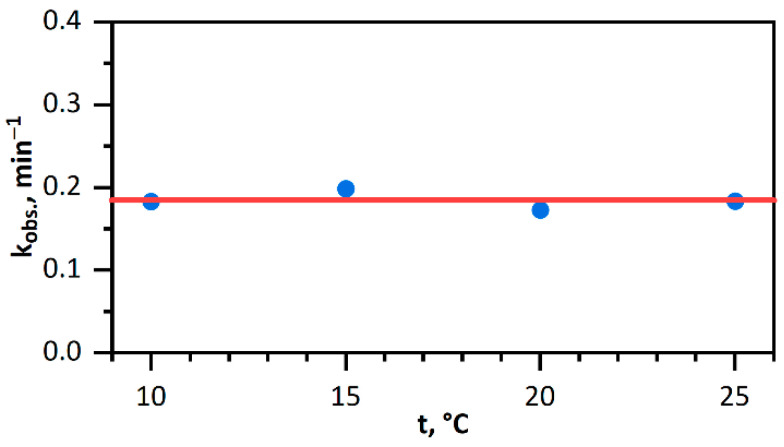
Observed rate constant of *Fe(CN)*_6_^3−^ reduction with hydrogen vs. temperature. Solution: [*Fe(CN)*_6_^3−^] = 1.00 mM; [*Pd*^0^] = 0.020 mM, [H_2_] = 0.80 mM (p(H_2_) = 1.025 atm).

Previously, it was noted that dissociative adsorption is a barrier-free process. To confirm the fact that dissociative adsorption is the slowest step, we studied the effect of temperature on the rate of catalytic reduction of hexacyanoferrate(III) ions with hydrogen in the presence of *Pd* NPs. It was shown (Figure 7) that in the 10 °C to 25 °C range, the reaction rate virtually does not depend on temperature to within the determination error. This phenomenon is attributable to the nearly zero activation energy of dissociative adsorption.

## 4. Conclusions

The results of our studies indicate that the catalytic activity of «hydrogenated» palladium is much higher than the catalytic activity of «non-hydrogenated» palladium. As the concentration of hydrogen dissolved in palladium (degree of «hydrogenation») increases, the rate of reduction of *Fe(CN)*_6_^3−^ ions with hydrogen substantially in-creases and at the limiting saturation these rates differ three to four-fold.

The accelerating effect of palladium hydrogenation can be attributed to the change in the palladium electronic state upon the dissolution of hydrogen. The formation of palladium hydride is likely to induce a considerable rearrangement of the electronic subsystem of the metal, which, as follows from experiments, activates the hydrogen dissociative adsorption on palladium particles and subsequent hydrogen atom ionization. The data of optical spectroscopy clearly indicate a change in the electronic state of *Pd* NPs upon the hydrogen saturation of an aqueous solution, possibly also caused by the formation of various forms of *Pd*-H [25,31]. The degree of hydrogen saturation determines the level of catalytic activity of palladium. The results also show that changing the temperature, pH, or stirring the solution does not affect the rate of the catalytic reaction. We assume that this is due to the fact that the limiting stage of the catalytic reaction is dissociative adsorption, rather than internal or external diffusion.

Palladium is among the most promising transition metals in catalysis and the results of this study can be useful for understanding of the mechanism of hydrogenation of organic compounds. Probably, they can be used to substantiate new approaches for optimization of palladium-catalyzed reactions of hydrogen. In particular, these approaches should take into account the important role of the electronic state of palladium, the catalytic activity of which is markedly affected by the amount of dissolved hydrogen and the possibility of varying the particle size and factors of the medium. In our opinion, an important line of future research would be elucidating the role of hydrogen dissolved in palladium in the selectivity of some organic synthesis reactions.

## Abbreviations

TEM	transmission electron microscopy
DLS	dynamic light scattering
PANa	polyacrilic acid sodium salt
Pd NPs	palladium nanoparticles
UV/Vis	ultraviolet and visible
UV	ultraviolet
